# The burden of dyslipidaemia and factors associated with lipid levels among adults in rural northern Ghana: An AWI-Gen sub-study

**DOI:** 10.1371/journal.pone.0206326

**Published:** 2018-11-28

**Authors:** Godfred Agongo, Engelbert Adamwaba Nonterah, Cornelius Debpuur, Lucas Amenga-Etego, Stuart Ali, Abraham Oduro, Nigel J. Crowther, Michèle Ramsay, as members of AWI-Gen and the H3Africa Consortium

**Affiliations:** 1 Navrongo Health Research Centre, Navrongo, Ghana; 2 Sydney Brenner Institute for Molecular Bioscience, Faculty of Health Sciences, University of the Witwatersrand, Johannesburg, South Africa; 3 Division of Human Genetics, National Health Laboratory Service and School of Pathology, Faculty of Health Sciences, University of the Witwatersrand, Johannesburg, South Africa; 4 Julius Global Health, Julius Center for Health Sciences and Primary Care, University Medical Centre Utrecht, Utrecht University, Utrecht, The Netherlands; 5 Department of Chemical Pathology, National Health Laboratory Service and School of Pathology, Faculty of Health Sciences, University of the Witwatersrand, South Africa; Loyola University Chicago, UNITED STATES

## Abstract

Dyslipidaemia is a primary risk factor for cardiometabolic disease, causing over 17 million deaths globally in 2015. However, the burden of dyslipidaemia and factors associated with lipid levels remain unknown in many rural African populations. Therefore, this study evaluated the association of socio-demographic, anthropometric and behavioural factors with lipid levels in rural Ghana. The prevalence of hypercholesterolaemia, hypertriglyceridaemia and elevated LDL-C in the total population of 1839 (846 men and 993 women) was 4.02%, 2.12%, and 5.55% respectively and did not differ between genders. The prevalence of low HDL-C levels was 60.30% and differed (p = 0.005) between men (56.86%) and women (63.24%). Subcutaneous abdominal fat was associated with TC (β = 0.067, p = 0.015) and TG (β = 0.137, p<0.001) among women and LDL-C (β = 0.139, p = 0.006) and TC (β = 0.071, p = 0.048) among men. Body mass index was associated with TC (β = 0.010, p = 0.043) among men while waist circumference was associated with LDL-C (β = 0.116, p<0.001) and TG (β = 0.094, p<0.001) among women. Hip circumference was negatively associated (β = -0.053, p = 0.043) while visceral fat was positively associated with TG (β = 0.033, p = 0.022) among women. Socioeconomic status, education, being unmarried and employment were associated with HDL-C (β = 0.081, p = 0.004), LDL-C (β = 0.095, p = 0.004) and TG (β = 0.095, p = 0.001) all among women, and TC (β = 0.070, p = 0.010) among men, respectively. Nankana women had lower TC (β = -0.069, p = 0.001), and men lower TG levels (β = -0.084, p = 0.008) than the other ethnic groups. Tobacco smoking (β = 0.066, p = 0.024) and alcohol intake (β = 0.084, p = 0.001) were associated with HDL-C levels among men and women respectively. Further studies are required to investigate whether high prevalence of low HDL-C levels in this population presents with any adverse cardiovascular disease outcomes. Associations of education, employment and adiposity with lipid levels suggest that future societal advances and increases in the prevalence of obesity may lead to associated adverse health consequences. Monitoring and interventions are required to limit these effects.

## Introduction

Dyslipidaemia is a metabolic derangement that predisposes an individual to atherosclerosis and cardiovascular disease (CVD) which caused over 17 million deaths globally in 2015, an increase of 12.5% from 2005 [1]. The contribution of dyslipidaemia to CVDs is evident from several longitudinal studies which have demonstrated the association of high levels of low density lipoprotein cholesterol (LDL-C), total cholesterol (TC), triglyceride (TG) and low levels of high density lipoprotein cholesterol (HDL-C) with CVD [2–5]. While dyslipidemia is progressively declining in developed and high-income countries, the same is not observed in middle- and low-income countries. Thus, it has been shown that TC levels decreased by 0.2mmol/l from the 1980s to 2008 in high-income countries, but declined by only 0.08–0.09mmol/l in middle- and low-income countries [6].

Serum lipid levels are reported to be influenced by anthropometric, demographic, environmental and genetic factors [7–10]. Consequently, the burden of dyslipidaemia in sub-Saharan Africa has been attributed mainly to urbanization which is associated with environmental, behavioural and socio-cultural changes [11,12]. In Ghana, previous studies have examined the burden of, and the factors associated with, dyslipidemia in mainly urban and semi-urban communities but few have evaluated the factors associated with serum lipid levels in rural settlements [13–17]. To date only one study has been conducted on lipid modulating factors in a rural settlement in Ghana but only the effect of anthropometric parameters was evaluated [17]. In rural communities in Northern Ghana where agriculture is the mainstay of the population [18] pesticide use is common. Findings elsewhere have associated pesticide use with increased lipid levels but this has not been evaluated to date in rural Ghana [19,20].

Just as urban settings in sub-Saharan Africa are experiencing a rising burden of dyslipidaemia and its adverse health consequences, rural areas are also facing the same challenge [21–23]. Treatment and control of dyslipidaemia in resource-poor settings is expensive [24] and will require innovative approaches for prevention, diagnosis and treatment. Measuring the prevalence and understanding the factors associated with dyslipidaemia in rural settings will assist in channeling scarce resources toward its prevention. This study therefore determined the burden of dyslipidaemia and evaluated how anthropometric, demographic and environmental factors are associated with serum lipid levels among adults in rural Northern Ghanaian communities.

## Materials and methods

### Study area

This study was carried out in the two Kassena-Nankana districts of north-eastern Ghana. The study area lies between latitude 10.30’ and 11.10’ north and longitude 1.1’ west, and covers a total land area of 1675km^2^, bordering northwards along the Ghana-Burkina Faso border. The area is characterized by Guinea Savannah vegetation dominated by semi-arid conditions and vast grassland integrated with short trees. The districts form the coverage area of the Navrongo Health and Demographic Surveillance System (NHDSS) which is implemented by the Navrongo Health Research Centre (NHRC) [25]. The study area is typical of many rural areas in sub-Saharan Africa where agriculture is the mainstay of the local economy with about 90% of the people being subsistence farmers. Poverty levels are high due to insufficient agricultural production resulting from a short rainy season and prolonged dry season [18]. The major ethnic groups in the area are the Kassena and Nankana, with several minority groups including the Bulsas [25].

### Study design and population

This population based cross-sectional study was conducted as part of the Africa Wits-INDEPTH Partnership for Genomic studies (AWI-Gen) project under the broader Human Heredity and Health in Africa (H3Africa) Initiative [26]. Participants were recruited from February to October 2015 in the west and east zones of the NHDSS area. These zones which are mainly Kassena and Nankana speaking communities respectively were purposefully selected for this study. A list of participants in the age range of 40 to 60 years was generated using the NHDSS database from each of the zones. A combined sample size of 2200 participants, including 10% for non-response or refusal to participate, of roughly equal gender ratio and with fairly equal representation from the major ethnic groups, was randomly selected. Of this number, 1050 participants were sampled from the east and 1150 participants were sampled from the west zones. Individuals who were resident within the study area for at least 10 years were recruited into the study after witnessed informed consent was obtained. Pregnant women were excluded from the study [26]. Participants who had no data for one or more of the investigated variables were excluded from the analytical data set. Therefore, a sample size of 1839 comprising 993 women and 864 men were included in the analysis.

### Data collection

Data on socio-demographic, anthropometric and behavioural factors were captured on a paper questionnaire [26] and uploaded into the Research Electronic Data Capture (REDCap) platform [27]. All participants were assigned a unique individual identification code on recruitment to ensure that both their identity, and data captured were anonymized. Data quality control included the checking of 10% of the entries for accurate data capture.

### Anthropometric and blood pressure measurements

Standing height and weight of each participant were measured without shoes and in light clothes using a Harpenden stadiometer (Holtain, Crymych, Wales) fixed to the wall and a digital calibrated weighing scale, respectively. Waist and hip circumference were measured using a non-stretch measuring tape (Seca GmbH& Co. KG, Hamburg, Germany) according to the guidelines of the WHO 2008 report on waist and hip circumference [28]. Body mass index (BMI), calculated as weight in kg/ height in m^2^, was categorized as underweight: <18.5kg/m^2^, normal weight:18.5–24.9kg/m^2^, overweight: 25.0–29.9kg/m^2^ and obese: ≥30kg/m^2^ according to WHO recommendation [29].

Visceral and abdominal subcutaneous fat tissue thicknesses were measured twice using a LOGIQ e ultrasound system with a 2–5.5 MHz 4C-RS curved transducer (GE, Healthcare, CT, USA) and the mean taken. The visceral fat thickness was defined as the distance in centimeters (cm) from the peritoneum to the vertebral bodies and subcutaneous fat thickness as the depth in cm from the skin to the linea alba. In order to visualize the relevant anatomical structures the scan depth was set at 15 cm for visceral fat and 9 cm for subcutaneous fat. The site for both measurements was where the xyphoid line and waist circumference meet.

Blood pressure was measured using a digital sphygmomanometer (Omron M6, Omron, Kyoto, Japan). The participant was seated on a chair and the feet firmly rested on the floor. The measurement was taken from the left hand which rested on a desk with the antecubital fossa level with the heart and palm facing upwards. The measurements were repeated twice more, with two minutes between each interval. The mean of the last two measurements were used to calculate the systolic blood pressure (SBP) and diastolic pressure (DBP) of the participant. High blood pressure was defined as SBP>140mmHg or DBP>90mmHg or self-reported controlled treatment using hypertensive medication [30].

### Biomarker analysis

Overnight fasting serum HDL-C, LDL-C, TG and TC were all measured directly using an automated chemistry analyzer (Randox RX Daytona+, Crumlin, Northern Ireland) at the University of the Witwatersrand Developmental Pathway for Health Research Unit (DHPRU), Chris Hani Baragwanath Hospital, Soweto, South Africa. A random selection of 150 samples were assayed in duplicate for glucose and all lipids to ascertain the coefficients of variation (CVs) of the assays. The CVs for glucose and the lipids (HDL-C, LDL-C, TC and TG) were 2.3% and less than 1.5%, respectively. Three control levels each were run for glucose and each of the lipid parameters. The mean bias relative to the reference standards [(measured mean- reference mean)/reference mean] for each of the levels for glucose was 0.12 for level 1, -0.03 for level 2 and 0.22 for level 3. The relative mean bias for each of the controls for HDL-C, LDL-C, TC and TG for levels 1, 2 and 3 respectively, were as follows: 0.07, 0.12 and 0.18; 0.14, 0.01 and -0.18; 0.05, 0.04 and -0.05; -0.27, -0.22 and -0.19, respectively.

All analyses were done in mmol/l, the equivalent in mg/dl being: mmol x 38.67 for HDL-C, LDL-C and TC; mmol/l x 88.57 for TG and mmol/l x 18 for glucose. Dyslipidaemia was defined as follows: high TC (>5.0mmol/l), high LDL-C (>3.0mmol/l), high TG (>1.7mmol/l), low HDL-C (<1.0mml/l for men and <1.2mmol/l for women) [31]. Participants needing treatment for hyperlipidaemia was defined according to the ATP III guidelines [32]. High blood glucose was defined as fasting serum glucose ≥7.0mmol/l [33].

### Socio-demographic and lifestyle variables

Self-reported data on ethnicity, marital status, employment, education, alcohol and tobacco use, household amenities, physical activity and pesticide exposure were categorized using the definitions described below.

Ethnicity was defined by self-reported ethnic origin or ancestral lineage. Marital status: a participant who was staying with a partner for at least a year during the period of recruitment was considered currently married. Employment status: a person, who reported to be involved in any form of employment, whether formal or informal, was considered employed. Education: this was defined by self-reported attainment of formal education. Alcohol consumption: data on alcohol consumption was collected using the CAGE questionnaire [34] that was adapted and incorporated into the main AWI-Gen questionnaire. A person was defined as having a current problematic drinking pattern if they reported ‘yes’ to more than two of the following: they felt they should cut down on their drinking, felt bad or guilty about their drinking, people have annoyed them by criticizing their drinking, and ever had an alcoholic drink first thing in the morning to steady their nerves or get rid of a hangover. Current non-problematic drinking pattern was defined as the drinking of alcohol by a person who took alcohol but reported affirmative to less than three of the above listed attributes. Tobacco use was defined as present or past consumption of cigarettes, pipes or smokeless tobacco.

Socioeconomic status: this was assessed using the INDEPTH Health Equity tool which is an asset index generated by using principal component analysis to combine data on household possessions (http://indepth-network.org/resources/indepth-health-equity-tool-measuring-socio-economic-status). The household assets were first broken down into categorical variables which were further converted into weights and principal components. The weights of the first principal component were used to develop an index from which scores of socioeconomic status of the households were derived. These scores were divided into quintiles with the first quintile being the poorest and the fifth the least poor. Physical activity was assessed using the Global Physical Activity Questionnaire (GPAQ) [35] which was incorporated into the main AWI-Gen questionnaire. Moderate to vigorous-intensity physical activity (MVPA) was calculated as minutes of physical activity per week (min/week). Pesticide exposure was defined by self-reported current working with pesticide or living close to a farm where pesticide was being used. Sleep duration was defined as the self-reported number of hours slept per night. Vegetable and fruit servings were estimated from self-reported quantities consumed per day using a serving size card. Malaria infection was defined as self-reported malaria fever within the past month. Menopausal status was categorized as follows: pre-menopausal status as having regular periods; menopausal transition as having irregular periods within the past 12 months; post-menopausal status as having no periods within the past 12 months [36].

### Ethical approval

The H3Africa AWI-Gen project was approved by the Human Research Ethics Committee (HREC) of the University of the Witwatersrand (ID No: M12109), the Ghana Health Service Ethics Review Committee (ID No: GHS-ERC:05/05/2015) and the Navrongo Institutional Review Board (ID No: NHRCIRB178). Community engagement was carried out prior to commencement of the study and signed or thump printed informed consent was obtained from each participant before being enrolled into the study.

### Statistical analyses

All statistical analyses were performed using STATA 14.2 (StataCorp, College Station, Texas, 77845, US). All continuous variables were presented as means with standard deviations and compared among men and women using Student’s non-paired t-test. Lipid levels were compared across age categories using ANOVA. Categorical variables were presented as percentages and compared between men and women using Pearson’s χ^2^ test. Awareness of dyslipidaemic or diabetes status was calculated using the number of participants who reported that they had been told by a health professional that they had high cholesterol or diabetes, respectively. Lipid levels were log-transformed to an approximate normal distribution and sex-stratified multivariable linear regression analyses were used to determine the association of each lipid species (dependent variable) with the selected study variables (independent variables). Variables that correlated with any of the lipids at p<0.20 in a univariate analysis were included in the multivariable linear regression model, and these models are displayed in the following tables. Multi-collinearity among variables was assessed using the variance inflation factor (VIF). All variables in the univariate analyses had VIF<5.0 and were therefore all included in the multivariable analyses. Residuals were approximately normally distributed and omitted variable bias was ruled out (p>0.05).

## Results

### Characteristics of study participants

A comparison of socio-demographic, behavioural, cardiometabolic and anthropometric variables across genders are presented in Table 1. The study population was 1839 participants and consisted of 54% women. The mean age (±SD) of the study participants was 51 ± 6 years. The majority ethno-linguistic groups were Kassena (51.93%) and Nankana (43.12%) with the remainder constituting the minority ethnic groups (Bulsa, Dagaati, Sisaala, Mampruga, Frafra, Hausa, and Akan). The population was mainly illiterate (69.87%) and a small proportion had either secondary (9.03%) or tertiary education (1.90%) with men constituting a greater proportion (p<0.001). Over half (62.91%) of the participants reported having some form of employment and more men were employed (p = 0.035) compared to women. Similarly, more men were least poor according to household attributes and were currently married compared to women (p<0.001 for both).

**Table 1:** Gender comparison of socio-demographic variables, food intake, exercise level, sleep duration, fasting blood glucose levels, blood pressure measurements and anthropometric variables.

Variables	Men	Women	Total	p-value
	(n = 846, 46%)	(n = 993, 54%)	(N = 1839)	Men vs women
**Age** (years)	50 ± 6.0	52 ± 6.0	51 ± 6.0	<0.001
**Ethnicity**				
Kassena	439 (51.89)	516 (51.96)	955 (51.93)	
Nankana	392 (46.34)	401 (40.38)	793 (43.12)	<0.001
Minority ethnic groups	15 (1.77)	76 (7.65)	91 (4.95)	
**Educational status**				
No formal education	517 (61.11)	768 (77.34)	1285 (69.87)	
Primary	192 (22.70)	161 (16.21)	353 (19.20)	<0.001
Secondary	111 (13.12)	55 (5.54)	166 (9.03)	
Tertiary	26 (3.07)	9 (0.91)	35 (1.90)	
**Employment status**				
Unemployed	292 (34.52)	390 (39.27)	682 (37.09)	
Employed	554 (65.48)	603 (60.73)	1157 (62.91)	<0.001
**Marital status**				
Currently married	717 (84.75)	632 (63.65)	1349 (73.36)	
Currently unmarried	129 (15.25)	361 (36.35)	490 (26.64)	
**Household SES categories**				
Poorest	129 (15.25)	204 (20.54)	333 (18.11)	
Very poor	139 (16.43)	192 (19.34)	331 (18.00)	
Poor	154 (18.20)	194 (19.54)	348 (18.92)	
Less poor	203 (24.00)	227 (22.86)	430 (23.38)	
Least poor	221 (26.12)	176 (17.72)	397 (21.59)	
**Fruit Intake** (servings/day)	1.01 ± 1.63	1.10 ± 1.69	1.06 ± 1.63	0.293
**Vegetable Intake**(servings/day)	3.43 ± 1.46	3.24 ± 1.51	3.33 ± 1.49	0.006
**Vendor meals** (times/week)	1.17 ± 1.68	0.79 ± 1.31	0.97 ± 1.50	<0.001
**MVPA**(hours/week)	40.07 ± 28.78	29.84 ± 27.72	34.55 ± 28.66	<0.001
**Sleeping** (hours/night)	7.71 ± 1.34	8.28 ± 1.32	8.02 ± 1.36	<0.001
**Fasting blood glucose**(mmol/l)	4.47 ± 0.76	4.61 ± 0.86	4.55 ± 0.82	<0.001
**SBP**(mmHg)	124.97 ± 20.44	123.28 ± 22.55	124.06 ± 21.61	0.094
**DBP**(mmol/l)	77.03 ± 12.86	77.12 ± 12.59	77.13 ± 12.72	0.760
**BMI** (kg/m^2^)	20.87 ± 3.15	22.28 ± 3.85	21.63 ± 3.61	<0.001
**Hip circumference** (cm)	8.41 ± 0.79	8.93 ± 0.99	8.69 ± 0.94	<0.001
**Waist circumference** (cm)	7.33 ± 0.81	7.68 ± 0.95	7.52 ± 0.91	<0.001
**Visceral fat** (cm)	4.18 ± 1.21	3.54 ± 1.12	3.83 ± 1.20	<0.001
**Subcutaneous fat** (cm)	0.78 ± 0.38	1.15 ± 0.54	0.98 ± 0.51	<0.001

Data is given as mean ± SD or n (%)

There was no difference between men and women in reported average fruit servings per day (p = 0.293) but reported vegetable servings per day (p = 0.006) and vendor meals per week (p<0.001) were significantly higher in men. The MVPA of the study population was 34.55 hours/week with men being more physically active than women (p<0.001). The mean sleep duration per night for men was significantly lower than that for women (p<0.001). Though not presented in the table, malaria infection in the past month was not significantly different between men and women (16.54% and 16.84% respectively; p = 0.868), and the prevalence of self-reported HIV positive cases in the study population was 0.93%. The mean fasting blood glucose of the study population was 4.55 ±0.82 mmol/l with that of women being significantly higher that of men (p<0.001). The mean values of SBP and DBP of the study population were 124.06 ± 21.61 mmHg and 77.13 ± 12.72 mmHg, respectively with no significant difference between men and women. All the anthropometric indices were higher in women than men (p<0.001) except visceral fat which was higher in men (p<0.001).

### Mean lipid levels stratified by sex and age category in the study population

**Fig 1:**
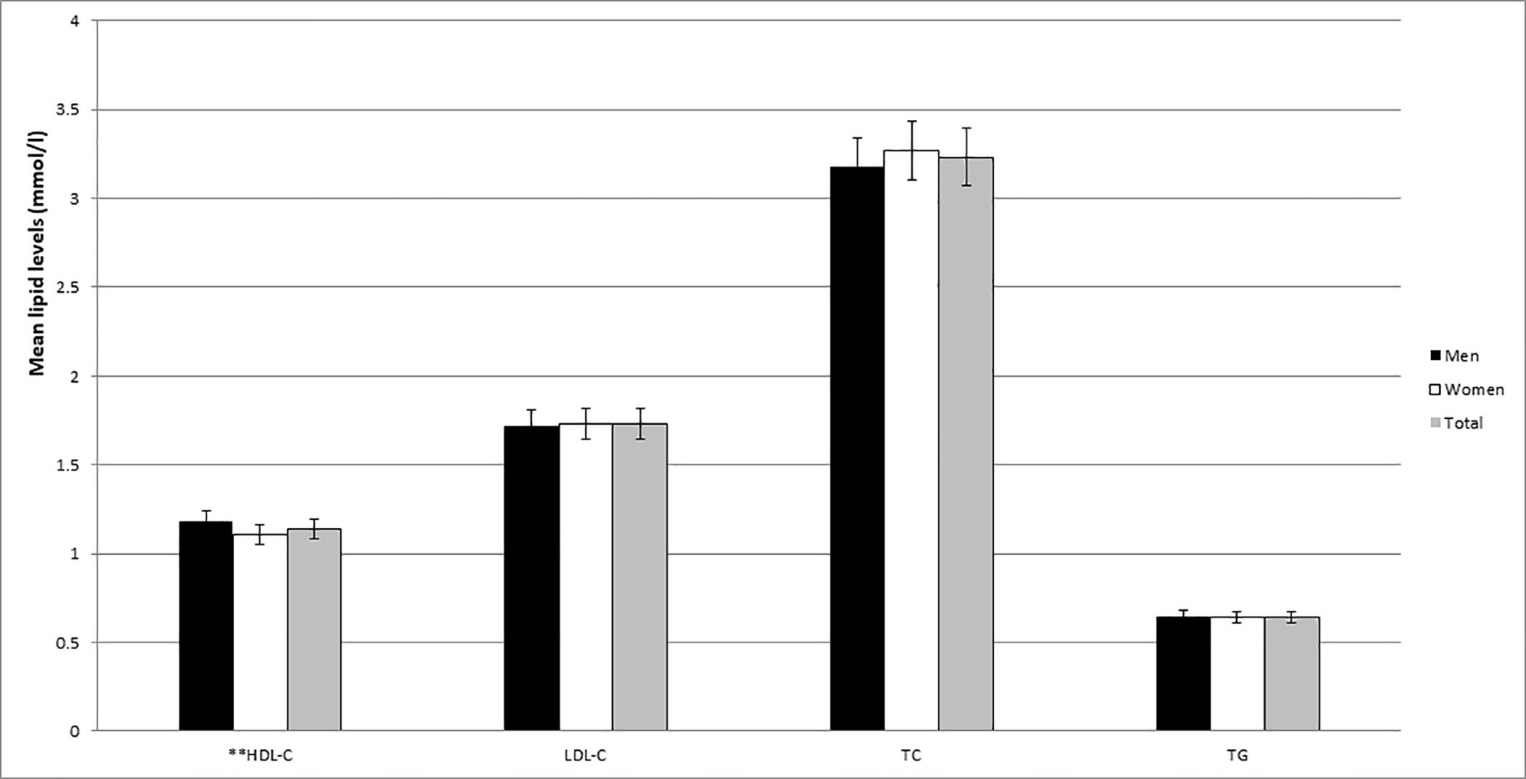
Mean lipid levels of the study population stratified by sex. **p<0.005 men vs women.

The mean HDL-C, LDL-C, TC and TG were 1.14 ± 0.38 mmol/l, 1.73 ± 0.78 mmol/l, 3.23 ± 0.92 mmol/l and 0.64 ± 0.45 mmol/l respectively. There were no significant differences in measured lipid levels between men and women except for HDL-C level which was significantly higher in men than in women (p = 0.001) (Fig 1).

The distribution of each lipid species in males and females is shown in the supplementary data files (see S1 Fig). The mean lipid levels were stratified according to the following age categories: 40–44 years, 45–49 years, 50–54 years and 55–60 years in males and females, and the data shown in the supplementary data files (see S1 Table). Only in females were correlations observed between age and lipids, in this case positive correlations of age with both TG (p = 0.006) and TC (p = 0.001). Significant gender differences were noted for HDL at age categories 45–49 and 55–60 years, with values higher in males than females. Males had significantly lower TC and TG than females in the age group 55–60 years.

### Prevalence of dyslipidaemia and other CVD risk factors in the study population stratified by sex

The prevalence of dyslipidaemia and other CVD risk factors among men and women is shown in Table 2. Smoking was far more common among men than women (p<0.001) but the use of smokeless tobacco did not differ between men and women (p = 0.997). Alcohol consumption was very prevalent with higher levels in males than females (p<0.001). Pesticide exposure was more common among men than women (p<0.001). Women were less physically active compared to men (p<0.001). The prevalence of overweight and obesity in the study population was 10.82% and 2.66% respectively with women being more overweight and obese than men (p<0.001). The prevalence of both high blood glucose (0.54%) and self-reported diabetes (0.76%) was very low with no difference between men and women (p = 0.309 and p = 0.529, respectively). The self-reported use of diabetes medication in the group with self-reported diabetes, was 50.0%, with no significant differences between genders (p = 0.515) There was no difference in the prevalence of high blood pressure between men and women (p = 0.464). The prevalence of hypercholesterolemia, hypertriglyceridaemia and elevated LDL-C in the total population was 4.02%, 2.12% and 5.55%, respectively. There were no significant sex differences for any of these sub-types of dyslipidaemia. However, the prevalence of low HDL-C concentration in the population was 60.30% and differed (p = 0.005) between men (56.86%) and women (63.24%). Awareness of dyslipidemia in this population was low at 0.38% and did not differ between women and men (p = 0.895). Only one person reported the use of lipid–lowering medication. The proportion of the population needing treatment for hyperlipidaemia was also low at (0.76%) with more men than women requiring treatment, but this difference just failed to reach statistical significance (p = 0.055). Pre-menopausal status among the women in the population was 37.44%, peri-menopausal status was 24.62% and the rest were in their post-menopausal status.

**Table 2:** Comparison across genders of the prevalence of smoking, alcohol intake, pesticide exposure, low physical activity, obesity, high blood sugar, high blood pressure, dyslipidaemia and required therapy for dyslipidaemia.

Variable	Men (n = 846, 46%)	Women (n = 993, 54%)	Total (N = 1839)	p-value
**Behavioural factors**				
Current smoker	185 (21.87)	14 (1.41)	199 (10.82)	<0.001
Former/current smokeless tobacco use	86 (10.17)	101 (10.17)	187 (10.17)	0.997
Former/current alcohol drinker	782 (92.43)	777 (78.25)	1559 (84.77)	<0.001
Pesticide exposure	509 (60.17)	497 (50.05)	1006 (54.70)	<0.001
Low physical activity	57 (6.74)	133 (13.39)	190 (10.33)	<0.001
**Body weight**				
Overweight	50 (5.91)	149 (15.01)	199 (10.82)	
Obese	8 (0.95)	41 (4.13)	49 (2.66)	<0.001
**Diabetes**				
High blood glucose	3 (0.35)	7 (0.70)	10 (0.54)	0.309
Self-reported diabetes	7 (0.84)	7 (0.70)	14 (0.76)	0.529
Self-reported diabetes medication	5 (71.42)	6 (85.71)	7 (50.00)	0.515
**High blood pressure**	190 (22.46)	209 (21.05)	399 (21.70)	0.464
**Dyslipidaemia**				
Low HDL-C	481 (56.86)	628 (63.24)	1109 (60.30)	0.005
High LDL-C	49 (5.79)	53 (5.34)	102 (5.55)	0.671
High TC	28 (3.31)	46 (4.63)	74 (4.02)	0.150
High TG	16 (1.89)	23 (2.32)	39 (2.12)	0.528
Self-reported dyslipidaemia	3 (0.35)	4 (0.40)	7 (0.38)	0.895
Self-reported dylipidaemia medication	0 (0.00)	1 (25.00)	1 (16.67)	0.439
Needing therapy for dyslipidaemia	10 (1.18)	4 (0.40)	14 (0.76)	0.055
**Menopausal status**				
Pre-menopausal	—	372 (37.44)	372 (37.44)	—
Peri-menopausal	—	245 (24.62)	245 (24.62)	—
Post-menopausal	—	376 (37.94)	376 (37.94_	—

Data is given as n (%)

### Factors associated with lipid levels in males and females

Due to the very low self-reported use of anti-diabetic and lipid-lowering medication, and the low prevalence of self-reported HIV infection, these variables were not included in the regression models developed for each of the lipid species.

The association of each of the study variables with LDL-C in both genders is illustrated in Table 3. In the univariate linear regression models formal education, high SES, BMI, waist circumference, hip circumference, visceral and subcutaneous fat were each associated with LDL-C among women. Age, formal education and waist circumference were associated with LDL-C in the multivariable regression model which explained only 8.2% (p<0.001) of the variance in LDL-C. In men the factors associated with LDL-C at the univariate level were formal education, high SES, vendor meals, sleep duration, BMI, waist circumference, hip circumference, visceral and subcutaneous fat. Only subcutaneous fat remained significant in the multivariable model which explained 5.1% (p<0.001) of the variance in LDL-C levels.

**Table 3:** Factors associated with LDL-C levels among men and women.

**Women**				
**Variable**	Univariate models		Multivariate model	
	β-Coefficient(95%CI)	p-value	β-Coefficient(95%CI)	p-value
Age(years)	0.004(-0.001, 0.009)	0.092	0.007(0.002, 0.012)	0.004
Nankana ethnicity	-0.040(-0.095, 0.016)	0.161	0.010(-0.046, 0.065)	0.735
Some formal education^1^	0.131(0.067, 0.196)	<0.001	0.095(0.030, 0.160)	0.004
High SES^2^	0.135(0.065, 0.206)	<0.001	0.026(-0.049, 0.100)	0.500
Used smokeless tobacco	-0.077(-0.167, 0.013)	0.092	-0.036(-0.124, 0.053)	0.433
Vendor (meals/week)	0.018(-0.003, 0.039)	0.085	0.013(-0.007, 0.034)	0.191
Sleeping (hours/night)	-0.019(-0.040, 0.001)	0.065	-0.010(-0.030, 0.011)	0.359
BMI (kg/m^2^)	-0.023(0.016, 0.030)	<0.001	-0.009(-0.023, 0.005)	0.201
Waist circumference (cm)	0.122(0.094, 0.149)	<0.001	0.116(0.060, 0.172)	<0.001
Hip circumference (cm)	0.089(0.062, 0.116)	<0.001	0.012(-0.032, 0.055)	0.596
Visceral fat (cm)	0.057(0.033, 0.081)	<0.001	0.001(-0.027, 0.028)	0.978
Subcutaneous fat (cm)	0.174(0.125, 0.223)	<0.001	0.046(-0.028, 0.119)	0.222
**Men**				
**Variable**	Univariate models		Multivariate model	
	β-Coefficient(95%CI)	p-value	β-Coefficient(95%CI)	p-value
Some formal education^1^	0.072(0.006, 0.138)	0.034	0.022(-0.045, 0.090)	0.514
Employed	0.056(-0.012, 0.124)	0.109	-0.001(-0.073, 0.073)	0.998
Currently unmarried	-0.061(-0.151, 0.029)	0.184	-0.014(-0.104, 0.075)	0.747
High SES^2^	0.148(0.075, 0.221)	<0.001	0.044(-0.037, 0.125)	0.285
Past or current smoker^3^	-0.045(-0.113, 0.023)	0.197	-0.004(-0.048, 0.040)	0.860
Past or current drinker^4^	-0.113(-0.235, 0.010)	0.071	-0.028(-0.157, 0.100)	0.663
Vegetable (servings/day)	0.020(-0.002, 0.042)	0.081	0.021(-0.003, 0.044)	0.087
Vendor (meals/week)	0.021(0.001, 0.040)	0.036	0.008(-0.012, 0.028)	0.419
Sleeping (hours/night)	-0.034(-0.058, -0.010)	0.006	-0.023(-0.047, 0.002)	0.067
BMI (kg/m^2^)	0.023(0.013, 0.033)	<0.001	0.001(-0.012, 0.014)	0.896
Waist circumference (cm)	0.114(0.074, 0.153)	<0.001	0.045(-0.011, 0.101)	0.117
Hip circumference (cm)	0.111(0.071, 0.151)	<0.001	0.032(-0.025, 0.088)	0.270
Visceral fat (cm)	0.034(0.007, 0.061)	0.013	0.001(-0.028, 0.030)	0.968
Subcutaneous fat (cm)	0.243(0.159, 0.326)	<0.001	0.139(0,040, 0.238)	0.006

CI: Confidence Interval; all lipid values were logged

^1^education was coded as some formal education vs. no education

^2^SES was coded as those with highest vs. those with lowest SES

^3^smoking status was coded as those who are current or past smokers vs. those who never smoked

^4^alcohol intake was coded as those who had ever drunk alcohol vs. those who had never drunk.

The principal modulators of HDL-C levels among men and women are shown in Table 4. High SES, past or current drinking and sleep duration were associated with HDL-C among women in the univariate analyses but only high SES and past or current drinking remained significant in the multivariable model. Only 2.3% (p<0.001) of the variance in HDL-C among women was explained by this model. In men, Nankana ethnicity, past or current smoking, past or current drinking and waist circumference were associated with HDL-C levels in the univariate analysis. Only past or current smoking remained associated with HDL-C in the multivariable model and explained only 0.95% (p = 0.062) of the variance in HDL-C levels.

**Table 4:** Factors associated with HDL-C levels stratified by sex.

**Women**				
**Variable**	Univariate models		Multivariate model	
	β-Coefficient(95%CI)	p-value	β-Coefficient(95%CI)	p-value
High SES^1^	0.077(0.022, 0.132)	0.006	0.081(0.025, 0.136)	0.004
Past or current smoker^2^	0.099(-0.018, 0.216)	0.096	0.087(-0.030, 0.203)	0.144
Used smokeless tobacco	0.060(-0.009, 0.129)	0.090	0.061(-0.008, 0.130)	0.084
Past or current drinker^3^	0.086(0.035, 0.136)	0.001	0.084(0.033, 0.134)	0.001
Sleeping (hours/night)	-0.016(-0.032, -0.001)	0.044	-0.013(-0.029, 0.003)	0.110
**Men**				
**Variable**	Univariate models		Multivariate model	
	β-Coefficient(95%CI)	p-value	β-Coefficient(95%CI)	p-value
Age(years)	0.004(-0.001, 0.008)	0.126	0.002(-0.003, 0.007)	0.463
Nankana ethnicity	0.054(0.003, 0.106)	0.039	0.041(-0.018, 0.100)	0.170
Past or current smoker^2^	0.081(0.028, 0.135)	0.003	0.066(0.009, 0.124)	0.024
Past or current drinker^3^	0.118(0.021, 0.214)	0.017	0.052(-0.053, 0.156)	0.334
Pesticide exposure	-0.043(-0.095, 0.010)	0.112	-0.005(-0.064, 0.055)	0.872
MVPA (hours/week)	0.001(-0.001, 0.002)	0.153	0.002(-0.001, 0.001)	0.796
Malaria in past month	-0.054(-0.125, 0.017)	0.133	-0.042(-0.114, 0.029)	0.243
BMI (kg/m^2^)	-0.007(-0.015, 0.001)	0.107	0.001(-0.011, 0.013)	0.876
Waist circumference (cm)	-0.033(-0.065, -0.001)	0.043	-0.029(-0.074, 0.017)	0.215
Hip circumference (cm)	-0.027(-0.060, 0.005)	0.102	0.006(-0.040, 0.052)	0.800

CI: Confidence Interval

^1^SES was coded as those with highest vs. those with lowest SES

^2^smoking status was coded as those who are current or past smokers vs. those who never smoked

^3^alcohol intake was coded as those who had ever drunk alcohol vs. those who had never drunk.

Table 5 shows the factors associated with TC levels among men and women. In the univariate regression models age, Nankana ethnicity, high SES, BMI, waist circumference, hip circumference and subcutaneous fat were each associated with TC level among women. However only age, Nankana ethnicity and subcutaneous fat remained significant in the multivariable regression model and these explained 4.9% (p<0.001) of the variance in TC. Among men SES, fruit consumption and BMI, were associated with TC concentration in the univariate models. Employment and BMI were significant in the multivariate analysis. This model explained 3.0% (p<0.001) of the variance in TC among men.

**Table 5:** Factors associated with TC among men and women.

**Women**				
**Variable**	Univariate models		Multivariate model	
	β-Coefficient(95%CI)	p-value	β-Coefficient(95%CI)	p-value
Age(years)	0.006(0.003, 0.009)	0.001	0.007(0.003, 0.010)	<0.001
Nankana ethnicity	-0.086(-0.126, -0.046)	<0.001	-0.069(-0.109, -0.029)	0.001
Currently unmarried	0.035(-0.006, 0.076)	0.095	0.025(-0.016, 0.066)	0.233
High SES^1^	0.055(0.004, 0.107)	0.035	0.027(-0.027, 0.081)	0.325
Sleeping (hours/night)	-0.012(-0.026, 0.003)	0.129	-0.008(-0.023, 0.007)	0.280
BMI (kg/m^2^)	0.013(0.006, 0.020)	0.001	-0.009(-0.019, 0.001)	0.083
Waist circumference (cm)	0.042(0021, 0.062)	<0.001	0.014(-0.026, 0.053)	0.493
Hip circumference (cm)	0.039(0.020, 0.059)	<0.001	0.030(-0.001, 0.062)	0.061
Subcutaneous fat (cm)	0.089(0.053, 0.125)	<0.001	0.067(0.013, 0.120)	0.015
**Men**				
**Variable**	Univariate models		Multivariate model	
	β-Coefficient(95%CI)	p-value	β-Coefficient(95%CI)	p-value
Employed	0.074(0.026, 0.122)	0.109	0.070(0.021, 0.118)	0.005
High SES^1^	0.063(0.011, 0.115)	0.018	0.011(-0.046, 0.068)	0.710
Used smokeless tobacco	-0.050(-0.126, 0.026)	0.197	-0.048(-0.124, 0.027)	0.210
Pesticide exposure	0.042(-0.005, 0.089)	0.079	0.018(-0.030, 0.066)	0.463
Fruit (servings/day)	0.021(0.007, 0.035)	0.004	0.016(0.002, 0.030)	0.059
BMI (kg/m^2^)	0.013(0.006, 0.020)	0.001	0.010(0.001, 0.019)	0.043

CI: Confidence Interval

^1^SES was coded as those with highest vs. those with lowest SES

Table 6 shows the factors associated with TG after stratifying by sex. Age, Nankana ethnicity, education, being unmarried, high SES, waist and hip circumferences, visceral and subcutaneous fat were all associated with TG in the univariate analyses among women. In the multivariate linear regression analysis age, being unmarried, waist and hip circumferences, visceral and subcutaneous adipose tissue thickness were significant. This model explained 12.0% (p<0.001) of the variance in TG levels. The factors associated with TG among men were Nankana ethnicity, high SES, use of smokeless tobacco, fruit consumption, waist circumference and visceral and subcutaneous fat thickness in the univariate analyses. In the multivariate analysis only Nankana ethnicity had a significant association with TG levels. This model accounted for 3.3% (p<0.001) of the variance in TG levels among men.

**Table 6:** Factors associated with TG among men and women.

**Women**					
**Variable**	Univariate models		Multivariate model	
	β-Coefficient(95%CI)	p-value	β-Coefficient(95%CI)	p-value
Age(years)	0.007(0.002, 0.012)	0.006	0.008(0.003, 0.012)	0.002
Nankana ethnicity	-0.075(-0.131, -0.018)	0.010	-0.013(-0.071, 0.045)	0.668
Some formal education^1^	0.097(0.031, 0.164)	0.004	0.046(-0.020, 0.113)	0.173
Currently unmarried	0.111(0.53, 0.168)	<0.001	0.095(0.037, 0.152)	0.001
High SES^2^	0.088(0.015, 0.160)	0.018	-0.010(-0.085, 0.066)	0.802
Vegetable (servings/day)	-0.017(-0.034, 0.003)	0.096	-0.012(-0.030, 0.006)	0.188
Malaria in past month	0.072(-0.004, 0.148)	0.062	0.055(-0.017, 0.127)	0.135
MVPA (hours/week)	-0.001(-0.002, 0.001)	0.090	-0.003(-0.001, 0.001)	1.000
Waist circumference (cm)	0.125(0.097, 0.153)	<0.001	0.094(0.043, 0.144)	<0.001
Hip circumference (cm)	0.068(0.041, 0.096)	<0.001	-0.053(-0.094, -0.011)	0.014
Visceral fat (cm)	0.083(0.059, 0.107)	<0.001	0.033(0.005, 0.062)	0.022
Subcutaneous fat (cm)	0.238(0.188, 0.287)	<0.001	0.137(0.065, 0.209)	<0.001
**Men**				
**Variable**	Univariate models		Multivariate model	
	β-Coefficient(95%CI)	p-value	β-Coefficient(95%CI)	p-value
Nankana ethnicity	-0.119(-0.174, -0.059)	<0.001	-0.084(-0.147, -0.022)	0.008
High SES^2^	0.110(0.041, 0.178)	0.002	0.041(-0.032, 0.115)	0.270
Used smokeless tobacco	-0.109(-0.209, -0.009)	0.032	-0.078(-0.177, 0.022)	0.125
Fruit (servings/day)	0.025(0.006, 0.043)	0.008	0.016(-0.002, 0.035)	0.085
Vendor (meals/week)	0.015(-0.003, 0.033)	0.095	0.007(-0.011, 0.025)	0.433
MVPA (hours/week)	-0.001(-0.001, 0.001)	0.090	-0.003(-0.001, 0.002)	0.347
Waist circumference (cm)	0.059(0.022, 0.096)	0.002	0.028(-0.016, 0.072)	0.207
Visceral fat (cm)	0.029(0.004, 0.054)	0.023	0.003(-0.024, 0.030)	0.844
Subcutaneous fat (cm)	0.152(0.074, 0.231)	<0.001	0.076(-0.016, 0.167)	0.105

CI: Confidence Interval

^1^education was coded as some formal education vs. no education

^2^SES was coded as those with highest vs. those with lowest SES

### Factors associated with lipid levels in total population

The association of factors with lipid levels in the combined population is shown in S2 Table for LDL and HDL and in S3 Table for TC and TG. All factors associated with lipid levels in either women or men or both show the same association in the combined population. The only exception is sleep duration which was associated with none of the lipid levels in the gender stratified analyses but was associated with LDL-C in the combined analyses. Interestingly, male gender was associated with higher HDL levels in the univariate analysis, but this association was lost in the multivariable model (S2 Table). This suggests that one of the variables in the multivariable analysis was confounding the gender effect. To isolate this variable we performed a univariate regression analysis of HDL with only gender included as an independent variable. We then systematically added each variable from the multivariable analysis into the model, one at a time, until we observed a variable that attenuated the gender effect to non-significance. The variable that did this was smoking. Smoking was more common in males than females (see Table 2) and correlated strongly with HDL levels in both the univariate and multivariate analyses (S2 Table). The relationship between smoking and HDL was investigated further to determine if other variables were modifying this association. The main variable chosen was alcohol intake, as this correlated with HDL in the univariate and multivariate models and was very prevalent in the population (see Table 2). In a univariate regression model for HDL, smoking correlated positively with the lipid in alcohol drinkers (β = 0.031, p<0.001, n = 1559), but in non-drinkers the relationship was negative but not significant (β = -0.014, p = 0.69, n = 280).

## Discussion

The current study shows that the prevalence of dyslipidaemia, as defined by high serum levels of TC, LDL-C or TGs, was relatively low (<6.0% for all 3 lipid species) in this rural Ghanaian community, and the proportion of subjects that needed treatment for high cholesterol was very low at 0.76%. However the prevalence of low HDL levels was very high at just over 60.0%. The major determinants of serum lipid levels, identified through multivariable linear regression analysis, were varied and included socio-demographic, behavioural and anthropometric factors. Ethnic grouping also influenced lipid levels with women and men from the Nankana ethnic group having lower TC and TG respectively than participants from the other ethnic groups.

The burden of dyslipidaemia in this population, as described by high LDL-C, TC or TG levels, was lower than that observed in Ghanaian urban settings [13,15] and lower than that noted in urban environments in other sub-Saharan African countries [12,37]. A previous study from Ghana comparing lipid profiles between urban and rural populations also demonstrated higher TC and LDL-C levels in the urban population, but higher TG levels in the rural cohort [15]. The higher prevalence of dyslipidaemia in urban settings may be due to reduced physical activity, westernization of diets and sedentary employment [38–40]. Our study also showed that very few dyslipidaemic participants were aware of their status. This may be due to the low background prevalence of hypercholesterolaemia and lack of access to appropriate medical facilities. A recent study in a rural South African population also showed a low level of awareness of dyslipidaemia [41].

Despite the low frequency of high TC, LDL-C and TG levels, we observed a very high prevalence of low HDL-C levels, as has also been observed in other studies from sub-Saharan Africa [42,43]. This raises the question of whether the current cut points used for diagnosing dyslipidaemia in African populations are appropriate, and whether the high prevalence of low HDL-C levels observed in this population suggest heightened CVD risk. One study has reported high rates of fatal CVD events in black African subjects with high HDL-C levels [44], and it has been shown that HDL-C levels in black participants are higher than in white participants even though the occurrence of CVDs is increasing more in the black population [45,46]. Furthermore, CVD risk is greater in subjects with low HDL in combination with high LDL or high triglyceride when compared to those with isolated low HDL levels [47]. This suggests that isolated HDL-C may not be a reliable indicator of CVD risk and supports the call for the shift of focus to HDL-C quality (HDL functionality and subclasses) rather than quantity [46,48] and the use of HDL in combination with other lipid species for predicting CVD risk [47].

Reports of sex differences in lipid levels in African populations are inconsistent. While some findings show that men have more favourable lipid profiles than women [23] other studies report no sex differences in lipids [49,50]. In this population men and women did not have significantly different lipid levels, with the exception of HDL-C which was lower in women. However, after adjusting for tobacco smoking within a multivariable linear regression model, the association between gender and HDL levels was no longer found to be significant. Furthermore, HDL levels correlated positively with smoking in males and in the total population. This is contrary to the literature, which consistently describes a negative relationship between these variables [51–53]. These data therefore suggested that the higher HDL levels in males were due to their higher frequency of tobacco smoking. Further analyses demonstrated that the positive relationship between smoking and HDL levels was only observed in alcohol drinkers, with the relationship being an inverse one (but not significant) in those who did not drink. This combined effect of smoking and alcohol intake on HDL levels is a novel finding, and requires further investigation in a larger sample size to assess the interaction between these variables and to disentangle the relative effects of smoking and alcohol intake on serum HDL levels. However, it must be noted that the positive association of smoking with HDL levels may be due to residual confounding. This problem may be minimized in future studies by using more objective measures of both smoking and possible confounding variables, including alcohol intake.

Our study did not show significant association between HDL-C levels and age. It has been suggested that low HDL-C is associated with age after 60 years [54]. Similarly studies have reported decreased HDL-C levels with age in cohorts with upper age ranges >60 years [55,56]. The lack of association of HDL-C with age in our study could possibly be due to the younger upper age range of our cohort. The positive association between age and LDL-C, TC and TG levels among women in this population reflects similar findings in previous studies [57,58]. The molecular mechanisms involved in the increase of serum cholesterol and TG levels with aging is not fully understood, although a number of possible causes have been suggested [59] including changes in hormone levels during menopause transition [60].

Lipid levels are known to show ethnic variation [61,62]. Our results show that the Nankana women have significantly lower TC and the Nankana men lower TG levels than their counterparts in the other ethno-linguistic groups analysed in this study. To our knowledge this is the first study in sub-Saharan Africa to assess differences in lipid levels between ethno-linguistic sub-groups within the same broad ethnic group (black Africans). This suggests possible genetic variability since the differences in lipid levels in these groups persist even after adjusting for anthropometric, behavioural and socio-demographic variables. However, these results must be confirmed in further studies that systematically compare lipid levels between these ethno-linguistic groups and adjust for a larger array of possible confounding factors.

The current study found a positive association of SES with HDL-C but a lack of association with LDL-C, TC and TG. This is contrary to several findings which indicate poorer lipid profiles with higher SES [63–65]. This may be due to the use of household assets in computing SES in our study rather than a combination of household assets, highest levels of education attained and employment status used in other studies [64,65].

There are mixed findings regarding the influence of employment status and education level on lipid levels. While some studies have associated low education levels and unemployment with higher lipid levels [65,66] others have reported the contrary [64,67,68]. The results of our study showed independent positive associations of formal education and employment with LDL-C among women and TC among men, respectively.

The positive association of alcohol consumption with HDL-C levels corroborate the findings of other studies [69,70]. It has been suggested that alcohol consumption may raise HDL-C levels by elevating the transport rate of the major apolipoproteins, Apo-I and Apo-II [71].

Pesticide exposure among the study population was high due to agricultural activities in the study area but there was no association between pesticide exposure and lipid levels [18]. This finding contradicts results of other studies which show a positive association of LDL-C, TC, TG and negative association of HDL-C levels with pesticide or chemical exposure [19,20]. The lack of association of pesticide exposure with lipid levels in our study may be due to the evaluation of the association of the exposure as opposed to the blood concentration of pesticides with lipid levels. Further studies are required to evaluate the association of pesticide concentration on lipid levels in this population.

There is a lack of consistent evidence regarding the influence of sleep duration on lipid levels. While some studies show an association of unfavourable lipid levels with increased night time sleep duration other studies show the contrary [72,73]. Though our study did not show association of sleep duration with any of the lipids in the gender stratified analysis, there was a negative association of sleep duration with LDL-C levels in the combined analysis. This calls for further studies on rural African adults to ascertain the influence of sleep duration on lipid levels.

Though diet (vendor meals, vegetable and fruit intake) is known to influence lipid levels our study did not find any association of lipid levels with diet. The lack of association between lipids and diet in our study may also be due to a lack of detailed information on daily food intake.

Despite the low level of obesity in this rural study, our results show that increased BMI was positively associated with TC among men and waist circumference was associated with both LDL-C and TG among women. Subcutaneous fat was associated with both TC and TG among women and both LDL-C and TC among men. The association of these markers of obesity with lipid levels are consistent with results of studies elsewhere [74–76]. Whilst visceral fat was associated with only TG, subcutaneous adipose tissue was associated with increasing LDL-C, TC and TG in the study population. These findings, unlike studies elsewhere that have indicated visceral adipose tissue as a better marker of dyslipidaemia [77,78], suggest that subcutaneous adipose tissue is an important factor influencing lipid levels in this population. Furthermore TG was negatively associated with hip circumference and this is consistent with other findings [77]. Hip circumference is a good measure of gluteofemoral fat which has been shown to protect against CVD risk factors such as insulin resistance, hypertension and dyslipidaemia [77,78].

## Conclusion

A longitudinal study is required to investigate whether high prevalence of low HDL-C really is a CVD risk factor in this community. The association of Nankana ethnicity with a healthier lipid profile in older adults is interesting and suggests that further studies, including genetic analyses, are required to understand this association. The association of education and employment with higher lipid levels suggests that societal advances will lead to increasing lipid levels in future, and so this must be monitored over time to allow early interventions. Likewise, the association of anthropometric variables with high lipid levels in a population with a low level of obesity suggests that any increase in the prevalence of obesity in this community may lead to high TC and TG levels. Hence, interventions should be developed to restrict this. Also, the association of hip circumference with lower TG levels suggests an attenuation effect of this adipose tissue depot at least on TGs but again this requires a longitudinal study to determine the magnitude of this effect.

### Strengths and limitations

This study has several strengths in terms of sample size, approach to biomarker analysis and the measurement of a large set of potential risk factors for dyslipidaemia. The sample of 1839 participants and the population-based study design is ideal for providing general baseline data. Many studies use derived LDL-C levels from the Friedewald equation, whereas we used a direct measurement method and implemented stringent quality control processes [75,79]. The study is anchored on the strength of the HDSS which provides an ideal platform for follow-up studies to establish the cause of dyslipidaemia among participants. Limitations are that participant age was often estimated based on an events calendar, and therefore may not be accurate, and lifestyle data were collected based on self-reported responses from the participants and could not be independently verified. The dietary data was limited and did not provide enough detail to truly establish whether food intake was associated with lipid levels. Finally, this was a cross-sectional study that could only investigate factors associated with lipid levels but could not establish causality. To further investigate the causes of abnormal lipid levels in the community the study will be extended to build a cohort that will allow for the collection of similar data from all participants five years after the baseline data collection.

## Supporting information

S1 Table Mean lipid levels of the study population stratified by sex and age category. Correlation performed using Pearson analysis; *p<0.05, **p<0.005 vs women.
(DOCX)

S2 Table Factors associated with LDL-C and HDL-C levels in the total population. (DOCX)

S3 Table Factors associated with TC and TG levels in the total population. (DOCX)

S1 Fig Distribution profiles for LDL-C, HDL-C, TC and TG in males and females. (DOCX)

S1 File Dataset. (CSV)
