# Correction: The burden of dyslipidaemia and factors associated with lipid levels among adults in rural northern Ghana: An AWI-Gen sub-study

**DOI:** 10.1371/journal.pone.0213233

**Published:** 2019-02-27

**Authors:** Godfred Agongo, Engelbert Adamwaba Nonterah, Cornelius Debpuur, Lucas Amenga-Etego, Stuart Ali, Abraham Oduro, Nigel J. Crowther, Michèle Ramsay, as members of AWI-Gen and the H3Africa Consortium

There are errors in the Hip circumference and Waist circumference values in Table 1. Please see the corrected Table 1 below.

**Table 1:** Gender comparison of socio-demographic variables, food intake, exercise level, sleep duration, fasting blood glucose levels, blood pressure measurements and anthropometric variables.

Variables	Men	Women	Total	p-value
	(n = 846, 46%)	(n = 993, 54%)	(N = 1839)	Men vs women
**Age** (years)	50 ± 6.0	52 ± 6.0	51 ± 6.0	<0.001
**Ethnicity**				
Kassena	439 (51.89)	516 (51.96)	955 (51.93)	
Nankana	392 (46.34)	401 (40.38)	793 (43.12)	<0.001
Minority ethnic groups	15 (1.77)	76 (7.65)	91 (4.95)	
**Educational status**				
No formal education	517 (61.11)	768 (77.34)	1285 (69.87)	
Primary	192 (22.70)	161 (16.21)	353 (19.20)	<0.001
Secondary	111 (13.12)	55 (5.54)	166 (9.03)	
Tertiary	26 (3.07)	9 (0.91)	35 (1.90)	
**Employment status**				
Unemployed	292 (34.52)	390 (39.27)	682 (37.09)	
Employed	554 (65.48)	603 (60.73)	1157 (62.91)	<0.001
**Marital status**				
Currently married	717 (84.75)	632 (63.65)	1349 (73.36)	
Currently unmarried	129 (15.25)	361 (36.35)	490 (26.64)	
**Household SES categories**				
Poorest	129 (15.25)	204 (20.54)	333 (18.11)	
Very poor	139 (16.43)	192 (19.34)	331 (18.00)	
Poor	154 (18.20)	194 (19.54)	348 (18.92)	
Less poor	203 (24.00)	227 (22.86)	430 (23.38)	
Least poor	221 (26.12)	176 (17.72)	397 (21.59)	
**Fruit Intake** (servings/day)	1.01 ± 1.63	1.10 ± 1.69	1.06 ± 1.63	0.293
**Vegetable Intake**(servings/day)	3.43 ± 1.46	3.24 ± 1.51	3.33 ± 1.49	0.006
**Vendor meals** (times/week)	1.17 ± 1.68	0.79 ± 1.31	0.97 ± 1.50	<0.001
**MVPA**(hours/week)	40.07 ± 28.78	29.84 ± 27.72	34.55 ± 28.66	<0.001
**Sleeping** (hours/night)	7.71 ± 1.34	8.28 ± 1.32	8.02 ± 1.36	<0.001
**Fasting blood glucose**(mmol/l)	4.47 ± 0.76	4.61 ± 0.86	4.55 ± 0.82	<0.001
**SBP**(mmHg)	124.97 ± 20.44	123.28 ± 22.55	124.06 ± 21.61	0.094
**DBP**(mmol/l)	77.03 ± 12.86	77.12 ± 12.59	77.13 ± 12.72	0.760
**BMI** (kg/m^2^)	20.87 ± 3.15	22.28 ± 3.85	21.63 ± 3.61	<0.001
**Hip circumference** (cm)	84.1 ± 7.9	89.3 ± 9.9	86.9 ± 9.4	<0.001
**Waist circumference** (cm)	73.3 ± 8.1	76.8 ± 9.5	75.2 ± 9.1	<0.001
**Visceral fat** (cm)	4.18 ± 1.21	3.54 ± 1.12	3.83 ± 1.20	<0.001
**Subcutaneous fat** (cm)	0.78 ± 0.38	1.15 ± 0.54	0.98 ± 0.51	<0.001

Data is given as mean ± SD or n (%)

There is an error in reference 14. The correct reference is: Asare-Anane H, Bawah AT, Osa-Andrews B, Adanu R, Ofori EK, Tagoe, Bani S A, Ateko EA R O, AK Nyarko. Lipid Profile In Ghanaian Women With Gestational Diabetes Mellitus. Int J Sci Technol Res. 2013;2(4):168–75.
