# Supplementary material for: The burden of dyslipidaemia and factors associated with lipid levels among adults in rural northern Ghana: An AWI-Gen sub-study
Source: PLoS One. 2018 Nov 28;13(11):e0206326. doi: 10.1371/journal.pone.0206326 (PMC6261546; doi:10.1371/journal.pone.0206326)
Supplement: S1 Table — Correlation performed using Pearson analysis; *p<0.05, **p<0.005 vs women. (DOCX) [file pone.0206326.s001.docx]

S1 Table: Mean lipid levels of the study population stratified by sex and age category

| **Lipids** | **Gender** | **40-44 years** | **45-49 years** | **50-54 years** | **55-60 years** | **R-value (p-value) for age vs lipid^1^** |
| --- | --- | --- | --- | --- | --- | --- |
| **HDL-C** | Men | 1.11 | 1.24** | 1.15 | 1.19** | 0.04 (0.13) |
|  | Women | 1.14 | 1.10 | 1.11 | 1.10 | -0.0001 (0.99) |
| **LDL-C** | Men | 1.65 | 1.82 | 1.67 | 1.72 | 0.02 (0.62) |
|  | Women | 1.66 | 1.73 | 1.66 | 1.80 | 0.05 (0.09) |
| **TC** | Men | 3.16 | 3.29 | 3.13 | 3.10** | -0.02 (0.59) |
|  | Women | 3.05 | 3.22 | 3.29 | 3.37 | 0.12 (0.001) |
| **TG** | Men | 0.62 | 0.69 | 0.68 | 0.60* | -0.02 (0.59) |
|  | Women | 0.61 | 0.62 | 0.62 | 0.67 | 0.09 (0.006) |

Correlation performed using Pearson analysis; *p<0.05, **p<0.005 vs women
