# Supplementary material for: The burden of dyslipidaemia and factors associated with lipid levels among adults in rural northern Ghana: An AWI-Gen sub-study
Source: PLoS One. 2018 Nov 28;13(11):e0206326. doi: 10.1371/journal.pone.0206326 (PMC6261546; doi:10.1371/journal.pone.0206326)
Supplement: S2 Table — (DOCX) [file pone.0206326.s002.docx]

S2 Table: Factors associated with LDL-C and HDL-C levels in the total population

| **LDL-C** | | | | |
| --- | --- | --- | --- | --- |
| **Variable** | Univariate models | | Multivariate model | |
|  | β-Coefficient(95%CI) | *P* value | β-Coefficient(95%CI) | P value |
| Age(years) | 0.003(-0.001, 0.007) | 0.105 | 0.006(0.002, 0.009) | 0.002 |
| Some formal education^1^ | 0.092(0.047, 0.138) | <0.001 | 0.059(0.013, 0.106) | 0.012 |
| Employed | 0.032(-0.011, 0.076) | 0.145 | 0.007(-0.036, 0.050) | 0.750 |
| High SES^2^ | 0.138(0.087, 0.188) | <0.001 | 0.043(-0.011, 0.036) | 0.118 |
| Used smokeless tobacco | -0.060(-0.129, 0.009) | 0.090 | -0.032(-0.100, 0.036) | 0.354 |
| Past or current smoker^3^ | -0.032(-0.077, 0.013) | 0.166 | 0.006(-0.027, 0.039) | 0.714 |
| Vendor (meals/week) | 0.018(-0.005, 0.032) | 0.009 | 0.012(-0.002, 0.025) | 0.099 |
| MVPA (hours/week) | -0.001(-0.001, 0.002) | 0.105 | -0.003(-0.001, 0.008) | 0.603 |
| Sleeping (hours/night) | -0.023(-0.039, -0.008) | 0.003 | -0.016(-0.032, -0.001) | 0.044 |
| BMI (kg/m^2^) | 0.023(0.017, 0.028) | <0.001 | -0.003(-0.012, 0.007) | 0.543 |
| Waist circumference (cm) | 0.118(0.094, 0.139) | <0.001 | 0.074(0.035, 0.112) | <0.001 |
| Hip circumference (cm) | 0.093(0.071, 0.115) | <0.001 | 0.022(-0.012, 0.056) | 0.211 |
| Visceral fat (cm) | 0.040(0.022, 0.057) | <0.001 | 0.003(-0.016, 0.022) | 0.760 |
| Subcutaneous fat (cm) | 0.177(0.137, 0.217) | <0.001 | 0.069(0.012, 0.125) | 0.017 |
| **HDL-C** | | | | |
| **Variables** | Univariate models |  | Multivariate model |  |
|  | β-Coefficient(95%CI) | *P* value | β-Coefficient(95%CI) | P value |
| Male gender | 0.047(0.014, 0.080) | 0.005 | -0.028(-0.074, 0.017) | 0.224 |
| Nankana ethnicity | 0.037(0.004, 0.070) | 0.027 | 0.023(-0.012, 0.057) | 0.119 |
| High SES^2^ | 0.037(-0.003, 0.077) | 0.068 | 0.057(0.015, 0.100) | 0.008 |
| Past or current smoker^3^ | 0.080(0.045, 0.116) | <0.001 | 0.077(0.030, 0.125) | 0.001 |
| Past or current drinker^4^ | 0.028(0.011, 0.045) | 0.001 | 0.080(0.032, 0.128) | 0.001 |
| MVPA (hours/week) | 0.001(-0.001, 0.002) | 0.153 | 0.003(-0.007, 0.001) | 0.535 |
| Sleeping (hours/night) | -0.010(-0.022, 0.002) | 0.096 | -0.007(-0.019, 0.006) | 0.304 |
| BMI (kg/m^2^) | -0.005(-0.009, -0.001) | 0.047 | -0.001(-0.008, 0.007) | 0.840 |
| Waist circumference (cm) | -0.019(-0.037, -0.001) | 0.041 | -0.015(-0.044, 0.015) | 0.341 |
| Hip circumference (cm) | -0.016(-0.034, 0.001) | 0.065 | 0.003(-0.024, 0.031) | 0.805 |

CI: Confidence Interval; ^1^education was coded as some formal education vs. no education; ^2^SES was coded as those with highest vs. those with lowest SES; ^3^smoking status was coded as those who are current or past smokers vs. those who never smoked; ^4^alcohol intake was coded as those who had ever drunk alcohol vs. those who had never drunk; R^2^=0.067 (p<0.001) for the LDL-C model and R^2^=0.019 (p<0.001) for the HDL-C model
