# Supplementary material for: The burden of dyslipidaemia and factors associated with lipid levels among adults in rural northern Ghana: An AWI-Gen sub-study
Source: PLoS One. 2018 Nov 28;13(11):e0206326. doi: 10.1371/journal.pone.0206326 (PMC6261546; doi:10.1371/journal.pone.0206326)
Supplement: S3 Table — (DOCX) [file pone.0206326.s003.docx]

S3 Table: Factors associated with TC and TG levels in the total population

| **TC** | | | | |
| --- | --- | --- | --- | --- |
| **Variables** | Unadjusted |  | Adjusted |  |
|  | β-Coefficient(95%CI) | P value | β-Coefficient(95%CI) | P value |
| Male gender | -0.034(-0.064, -0.004) | 0.027 | -0.009(-0.051, 0.034) | 0.679 |
| Age(years) | 0.003(0.001, 0.006) | 0.018 | 0.004(0.002, 0.007) | 0.002 |
| Nankana ethnicity | -0.093(-0.123, -0.063) | <0.001 | -0.079(-0.111, -0.046) | <0.001 |
| Employed | 0.030(-0.001, 0.061) | 0.062 | 0.035(0.004, 0.066) | 0.028 |
| Currently unmarried | 0.024(-0.010, 0.058) | 0.170 | 0.018(-0.017, 0.053) | 0.311 |
| High SES^2^ | 0.055(0.018, 0.091) | 0.003 | 0.025(-0.014, 0.063) | 0.212 |
| Past or current smoker^3^ | -0.021(-0.054, 0.011) | 0.192 | 0.021(-0.021, 0.064) | 0.372 |
| Pesticide exposure | 0.026(-0.004, 0.051) | 0.090 | 0.003(-0.029, 0.035) | 0.862 |
| BMI (kg/m^2^) | 0.010(0.006, 0.014) | 0.001 | 0.001(-0.006, 0.007) | 0.873 |
| Waist circumference (cm) | 0.038(0.022, 0.055) | <0.001 | -0.004(-0.032, 0.024) | 0.776 |
| Hip circumference (cm) | 0.038(0.022, 0.054) | <0.001 | 0.014(-0.011, 0.039) | 0.278 |
| Subcutaneous fat (cm) | 0.094(0.065, 0.124) | <0.001 | 0.063(0.020, 0.105) | 0.004 |
| **TG** | | | | |
| **Variables** | Univariate models |  | Multivariate model |  |
|  | β-Coefficient(95%CI) | P value | β-Coefficient(95%CI) | P value |
| Age(years) | 0.003(-0.001, 0.007) | 0.105 | 0.004(0.001, 0.008) | 0.025 |
| Nankana ethnicity | -0.095(-0.136, -0.054) | <0.001 | -0.047(-0.092, -0.003) | 0.036 |
| Some formal education^1^ | 0.060(0.015, 0.104) | 0.009 | 0.022(-0.024, 0.069) | 0.343 |
| Currently unmarried | 0.064(0.018, 0.111) | 0.006 | 0.062(0.015, 0.109) | 0.010 |
| High SES^2^ | 0.099(0.049, 0.148) | <0.001 | 0.029(-0.025, 0.082) | 0.288 |
| Used smokeless tobacco | -0.081(-0.148, -0.013) | 0.019 | -0.051(-0.119, 0.017) | 0.114 |
| MVPA (hours/week) | -0.001(-0.003, -0.001) | 0.026 | -0.006(-0.001, 0.001) | 0.991 |
| Sleeping (hours/night) | -0.011(-0.026, 0.004) | 0.142 | -0.007(-0.021, 0.009) | 0.415 |
| Vegetable (servings/day) | -0.014(-0.028, -0.001) | 0.046 | -0.007(-0.022, 0.007) | 0.317 |
| Vendor (meals/week) | 0.012(-0.002, 0.026) | 0.084 | 0.007(-0.008, 0.013) | 0.362 |
| Malaria in past month | 0.058(0.002, 0.114) | 0.044 | 0.043(-0.012, 0.098) | 0.125 |
| BMI (kg/m^2^) | 0.019(0.014, 0.025) | <0.001 | 0.003(-0.007, 0.013) | 0.600 |
| Waist circumference (cm) | 0.100(0.073, 0.118) | <0.001 | 0.075(0.036, 0.114) | <0.001 |
| Hip circumference (cm) | 0.042(0.020, 0.064) | <0.001 | -0.074(-0.107, -0.040) | <0.001 |
| Visceral fat (cm) | 0.053(0.036, 0.070) | <0.001 | 0.028(0.010, 0.047) | 0.003 |
| Subcutaneous fat (cm) | 0.183(0.143, 0.222) | <0.001 | 0.123(0.067, 0.179) | <0.001 |

CI: Confidence Interval; ^1^education was coded as some formal education vs. no education; ^2^SES was coded as those with highest vs. those with lowest SES; ^3^smoking status was coded as those who are current or past smokers vs. those who never smoked; R^2^=0.038 (p<0.001) for the TC model and R^2^=0.073 (p<0.001) for the TG model
