# Supplementary material for: The burden of dyslipidaemia and factors associated with lipid levels among adults in rural northern Ghana: An AWI-Gen sub-study
Source: PLoS One. 2018 Nov 28;13(11):e0206326. doi: 10.1371/journal.pone.0206326 (PMC6261546; doi:10.1371/journal.pone.0206326)
Supplement: S1 Fig — (DOCX) [file pone.0206326.s004.docx]

**S1 Figure: Distribution profiles for LDL-C, HDL-C, TC and TG in men and women**
